# BSTA: a targeted approach combines bulked segregant analysis with next- generation sequencing and *de novo* transcriptome assembly for SNP discovery in sunflower

**DOI:** 10.1186/1471-2164-14-628

**Published:** 2013-09-17

**Authors:** Maren Livaja, Yu Wang, Silke Wieckhorst, Grit Haseneyer, Michael Seidel, Volker Hahn, Steven J Knapp, Stefan Taudien, Chris-Carolin Schön, Eva Bauer

**Affiliations:** 1 Plant Breeding, Technische Universität München, 85354 Freising, Germany; 2 Helmholtz Zentrum München, Institute of Bioinformatics and Systems Biology/MIPS, 85764 Neuherberg, Germany; 3 State Plant Breeding Institute, Universität Hohenheim, 70599 Stuttgart, Germany; 4 Center for Applied Genetic Technologies, University of Georgia, Athens, GA 30602 USA; 5 Leibniz Institute for Age Research, Fritz Lipmann Institute, 07745 Jena, Germany; 6 Present address: KWS SAAT AG, 37555 Einbeck, Germany; 7 Present address: Monsanto Vegetables, Inc, Woodland, CA 95695, USA

**Keywords:** Bulked segregant transcriptome analysis, 454 next-generation sequencing, Marker enrichment pipeline, *De novo* transcriptome assembly, Resistance gene candidates, *Helianthus argophyllus*, *Helianthus annuus*, Sunflower, *Plasmopara halstedii*, *Pl*_*ARG*_

## Abstract

**Background:**

Sunflower belongs to the largest plant family on earth, the genomically poorly explored *Compositae*. Downy mildew *Plasmopara halstedii* (Farlow) Berlese & de Toni is one of the major diseases of cultivated sunflower (*Helianthus annuus* L.). In the search for new sources of downy mildew resistance, the locus *Pl*_*ARG*_ on linkage group 1 (LG1) originating from *H. argophyllus* is promising since it confers resistance against all known races of the pathogen. However, the mapping resolution in the *Pl*_*ARG*_ region is hampered by significantly suppressed recombination and by limited availability of polymorphic markers. Here we examined a strategy developed for the enrichment of molecular markers linked to this specific genomic region. We combined bulked segregant analysis (BSA) with next-generation sequencing (NGS) and *de novo* assembly of the sunflower transcriptome for single nucleotide polymorphism (SNP) discovery in a sequence resource combining reads originating from two sunflower species, *H. annuus* and *H. argophyllus*.

**Results:**

A computational pipeline developed for SNP calling and pattern detection identified 219 candidate genes. For a proof of concept, 42 resistance gene-like sequences were subjected to experimental SNP validation. Using a high-resolution mapping population, 12 SNP markers were mapped to LG1. We successfully verified candidate sequences either co-segregating with or closely flanking *Pl*_*ARG*_.

**Conclusions:**

This study is the first successful example to improve bulked segregant analysis with *de novo* transcriptome assembly using next generation sequencing. The BSTA pipeline we developed provides a useful guide for similar studies in other non-model organisms. Our results demonstrate this method is an efficient way to enrich molecular markers and to identify candidate genes in a specific mapping interval.

## Background

Downy mildew caused by the oomycete *Plasmopara halstedii* (Farlow) Berlese & de Toni is responsible for significant yield losses during sunflower cultivation. Interspecific hybridization between *Helianthus* species is an important tool to expand the genetic variability of cultivated sunflower and to extend the genetic basis of disease resistance in breeding material [1,2]. The downy mildew resistance locus *Pl*_*ARG*_ was introgressed from the wild species *H. argophyllus* into cultivated sunflower [3]. Since it mediates resistance to all known races of *P. halstedii*, *Pl*_*ARG*_ is a valuable source for broad-spectrum resistance against the pathogen. Recently, we have fine-mapped the *Pl*_*ARG*_ locus on linkage group 1 (LG1) of sunflower [4], but the ultimate goal is the positional cloning of the resistance gene or gene cluster underlying *Pl*_*ARG*_. For this purpose, we devised a strategy for the enrichment of that region with markers by focusing on a subset of sequences with homology to known plant resistance genes. The identification of resistance gene-like sequences located in the target interval thus serves as proof-of-concept for the employed enrichment strategy and provides candidate sequences for the resistance gene *Pl*_*ARG*_.

The approach used for marker enrichment in non-model organisms generally depends on the availability of genomic information. To date no reference sequence is available for sunflower. Whole genome sequencing and the establishment of a reference sequence of *H. annuus* is advancing rapidly, however, due to its genome size of about 3.5 Gb and its highly repetitive nature sequencing the sunflower genome is a time- and cost-intensive enterprise even with NGS technologies [5]. For identification of candidate genes derived from interspecific crosses it remains to be shown how informative this sequence information will be. In case of *Pl*_*ARG*_, a large chromosome segment from *H. argophyllus* was introgressed into LG1 of *H. annuus*, but as for many other crop species, no reference sequences will be available for the wild relatives of sunflower in the near future. Thus, for the detection of candidate genes a sequence by sequence comparison will not be possible. In addition, the alien genome introgression was accompanied by suppressed recombination around *Pl*_*ARG*_ that was reflected by the clustering of SSR markers on LG1 [4]. Therefore, extremely large mapping populations are required to recover informative crossovers in the target region necessary for constructing high-resolution genetic maps.

As an efficient method for the rapid identification of molecular markers linked to any specific gene or genomic region, Michelmore *et al.*[6] developed bulked segregant analysis (BSA). The idea of BSA is to establish two phenotypically contrasting bulked samples that contain individuals of a population segregating for the gene of interest. The individuals within one bulk carry the same allele for one particular gene or genomic region surrounding the gene of interest, but carry arbitrary alleles at all unlinked regions. Similar to near-isogenic lines, BSA can efficiently be used for marker enrichment in a target region [7].

NGS technologies have been used for a wide range of plant genomic applications such as genome sequencing in cucumber [8], *de novo* sequencing of BACs in barley [9], whole-genome sequence variation studies on *A. thaliana* accessions [10] and transcriptome sequencing for SNP discovery in rye [11]. Applying high-throughput sequencing to BSA in yeast as a species with a rather small genome, Wenger *et al.* discovered a xylitol dehydrogenase gene responsible for xylose utilization [12]. Recently, Trick *et al.* reported the successful combination of BSA and Illumina RNAseq technology for fine-mapping of the previously cloned grain protein content gene *GPC-B1* in tetraploid wheat. The SNP discovery was realized by aligning individual reads against a NCBI wheat transcriptome reference comprising 40,349 unigene sequences [13].

Here, we describe a procedure for marker enrichment and candidate gene identification that uses NGS in combination with a Bulked Segregant Transcriptome Analysis (BSTA) approach. As only a small percentage of the genome constitutes the transcriptome, deep sequencing of cDNA libraries provides an attractive approach to obtain sufficient sequence coverage needed for *de novo* assembly and for discovery of SNP markers. Main objectives of this study were to 1) develop a SNP detection pipeline integrating bulked segregant analysis with *de novo* transcriptome assembly, 2) enrich for SNP markers in the target region around the *Pl*_*ARG*_ locus, and 3) verify the performance of our *in silico* analysis by experimental SNP validation. For validation of our method, we chose a subclass of sequences related to known disease resistance genes in plants, because the number of detected sequence polymorphisms between the two bulks was very large and this class of genes was most suitable for the identification of candidate genes for our target locus *Pl*_*ARG*_.

To our knowledge, this is the first study describing SNP discovery in a non-model plant based on NGS transcriptome sequencing using two phenotypically and genotypically contrasting bulks and combined with *de novo* assembly. We show the feasibility of the BSTA approach for efficient marker enrichment in a specific target region of a non-model organism. Our approach for marker enrichment and candidate gene identification in a target region can be extended to any other species with or without reference genome.

## Methods

### Plant material, bulk formation and cDNA synthesis

A segregating population comprising 2,141 F_2_ individuals was developed using (cms)HA342 as downy mildew susceptible parent and ARG1575-2 as resistance donor [4]. HA342 is derived from a single BC_1_F_4_ plant from the cross HA89*2/Pervenets. ARG1575-2 is an inbred line derived by crossing *H. argophyllus* accession 1575 (PI 468651) with cmsHA89 followed by two generations of backcrossing with cmsHA89 and five selfing generations. It donates resistance to *P. halstedii* through the introgression of a *H. argophyllus* segment on LG1.

Resistance to *P. halstedii* was evaluated after inoculating plants with downy mildew spores (race 730, 710, 100, and 330). Phenotyping of F_2:3_ families derived from (cms)HA342xARG1575-2 identified homozygous susceptible and homozygous resistant F_2_ plants [4]. In addition, molecular marker screening determined the genotypic state of the phenotyped plants in the target interval of 0.3 cM between the previously mapped microsatellite markers ORS509 and HT446 on LG1. Two phenotypically contrasting bulks, one resistant (BR) and one susceptible (BS) to *P. halstedii* and each comprising 16 F_2_ plants were generated. F_2_ plants that 1) showed a resistant or susceptible phenotype, 2) were homozygous in the target interval as determined by the microsatellite marker genotypes and 3) carried a recombination event above or below the target region were pooled (see Additional files 1: Table S1 and Additional file 2: Table S2). This pooling strategy avoided heterozygous segments in the target window and delimited the target interval as much as possible.

Under the assumption that candidate genes for *Pl*_*ARG*_ are constitutively expressed at a weak level in non-inoculated plants [14,15], seedlings were grown in the greenhouse at a 14 h light (18°C)/10 h dark (16°C) cycle for eight days. Hypocotyls, cotyledons, and leaves were harvested separately, immediately frozen in liquid nitrogen and stored at −80°C. Tissue-specific RNA extraction was performed using the NucleoSpin® RNA Plant Kit (#740949.50, Macherey-Nagel, Düren, Germany) according to the manufacturer’s protocol. Equal amounts of hypocotyl, cotyledon and leaf RNA from the selected 16 F_2_ plants were pooled separately for BR and BS. cDNA synthesis, normalization, size fractionation and sequencing adapter ligation were performed by vertis Biotechnologie AG (Freising, Germany). For each bulk, a specific hexamer sequence tag (BR: ATACTG, BS: GATAGC) was linked to the 5′-sequencing adapter allowing for filtering of reads after sequencing. Normalized, size-fractioned, and adapter-ligated double stranded cDNAs of BR and BS were pooled and 200 ng was used for 454 sequencing.

### BSTA pipeline

#### Next generation transcriptome sequence analysis

**Figure 1:**
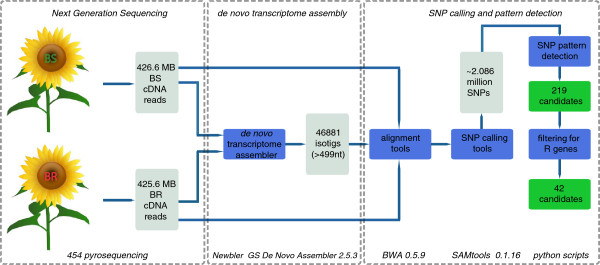
**Bulked segregant transcriptome analysis (BSTA) data processing pipeline.** Using next generation cDNA sequencing of one susceptible bulk (BS) and one resistant bulk (BR), *de novo* transcriptome assembly, SNP calling and pattern detection, and the filtering for *R* gene like sequences resulted in 42 candidates for verification. Respective technologies and tools used in our study are listed at the bottom.

Our bulked segregant transcriptome analysis (BSTA) data processing pipeline consists of three major steps (Figure 1), which are further divided into several components. The first step of the pipeline is the use of next generation sequencing technology to generate transcriptome sequences of sunflower. For the *de novo* transcriptome sequencing we chose Roche 454 pyrosequencing because it generates longer reads as compared to the Illumina technology [16]. Sequencing of the BR and BS cDNA pool was carried out according to the manufacturer’s instructions (GS FLX Titanium General library preparation kit/emPCR kit/sequencing kit, Roche Diagnostics, http://www.roche.com). cDNA synthesis primer and sequencing adapter sequences were trimmed from raw 454 sequence reads. The two sequence libraries of the BR and BS bulks were filtered by removing sequences that were either shorter than 50 nt or of low sequencing quality. The removal of shorter sequences was carried out using a custom PYTHON script. The filtering of low quality sequences was done by Newbler GS De Novo Assembler 2.5.3 (Roche, Branford, CT, USA) automatically before starting the assembling process.

#### *De novo* transcriptome assembly

The second step of the BSTA pipeline was to create a reference sequence set against which SNP calling could be carried out (Figure 1). This step is necessary if no reference genome is available, as is the case in sunflower. If a reference genome is available, reads of the contrasting bulks can directly be mapped against the reference sequence as described in the next paragraph. The two sequence libraries of BR and BS bulks were pooled in order to increase coverage and reliability of the reference sunflower transcriptome, which was *de novo* assembled by the Newbler GS De Novo Assembler. Incremental *de novo* assembly analysis was performed with a parameter setting requiring a minimum sequence overlap length of 40 nt, a minimum overlap identity of 90% and using 16 CPU cores. During the transcriptome assembling process, Newbler builds a contig graph where the reads coming from the transcript of a certain gene should be assembled into a single contig. However, splice-variants of a single gene may cause a break of the contig graph (Additional file 3: Figure S1). In transcriptome assemblies, all transcript variants (subgraphs), each potentially representing the same gene, are collected together and named “isogroup” by Newbler. The transcript variants within one isogroup are referred to as isotigs which represent alternative splice-variants, whereas the contigs represent the exons, see Additional file 3: Figure S1 [17]. In order to identify bulk specific contigs, BR and BS reads were assembled together but the information about the read origin was retained. The developed sunflower transcriptome reference assembly, hereafter called BRBS, was then filtered to retain isotigs that were longer than 499 nt. At positions where sequence variants were observed, the major allele was considered as the reference allele. The established sunflower sequence resource comprising the raw sequence data and the assembly is available from the GABI primary database (http://www.gabipd.org) [18,19].

#### SNP calling and pattern detection in BR and BS

During the third major step of the pipeline, BR and BS reads were aligned independently to the isotigs of the transcriptome reference sequence (Figure 1). For the application of Burrows-Wheeler Alignment Tool (BWA), reads were separated into a short read set (50 nt to 199 nt) and a long read set (> 199 nt). The short read set was aligned using BWA 0.5.9 [20] and the long read set was aligned using BWA-SW (from BWA 0.5.9 software package) [21]. Alignment results of the short and long read sets from the same bulk were merged by Sequence Alignment/Map tools (SAMtools 0.1.16) [22]. Duplicate 454 reads were removed by SAMtools. Pileup files were generated by SAMtools to facilitate SNP calling. In the pileup files each line represents a sequence position of the reference sequences, consisting of the sequence name, position, reference base, number of reads covering the site, read bases and base qualities. Following earlier transcriptome-based studies for SNP discovery in plants [23-25] SNPs with a coverage ≥ 5 reads per bulk were selected for further SNP pattern detection. For each SNP in one bulk, the corresponding sequence position was checked in the pileup file from the other bulk to see if, 1) the coverage from the other library was ≥ 5, and 2) all the aligned reads from the other bulk had the reference nucleotide at this position. All SNP containing sequences that fulfilled these criteria were defined as putative candidate genes.

All computational steps necessary to connect the different software tools for SNP calling and pattern detection were performed using custom PYTHON scripts.

After SNP pattern detection, candidate isogroups were annotated using Blast2GO [26]. In order to identify transcripts involved in disease resistance, tblastx analysis (E-value cutoff = 1E-10) was performed to detect similarity to 113 plant resistance genes from the Plant Resistance Genes database (http://prgdb.cbm.fvg.it/index.php, accessed April, 2011) [27]. Finally, from each resistance-gene-like isogroup one isotig was randomly selected for experimental SNP validation.

### SNP validation and genetic mapping

For confirmation of identified SNP polymorphisms and their subsequent genetic mapping, two genotyping systems were adopted. Genotyping of isotig sequences in F_2_ individuals of the (cms)HA342 x Arg1575-2 population was performed by KBioscience Ltd (http://www.kbioscience.co.uk) by applying the KASPar chemistry. Isotig sequences for which the *in silico* test for KASPar assay design was not successful were used to develop cleaved amplified polymorphic sequence (CAPS) markers [28]. For Sanger re-sequencing of selected isotigs, SNP flanking primers were designed using BatchPrimer3 [29]. PCR amplification was carried out using DNA of the parental lines HA342 and ARG1575-2 as template. The final volume was set to 10 μl containing 275 nM of each primer, 0.4 U *Taq* DNA polymerase (Q-Biogene, Illkirch, Germany), 1x *Taq* DNA polymerase buffer, 2.0 mM MgCl_2_, and 0.2 mM of each dNTP. Amplicons were sequenced in both directions using the specific PCR primers as sequencing primers and the ABI Prism BigDye® v1.1 Cycle Sequence Kit (#4337450, Applied Biosystems, Foster City, CA, USA) on an ABI3130 following manufacturer’s instructions. Raw data were analyzed using software Sequencing Analysis v5.3.1 (Applied Biosystems) and Sequencher v5.0 (Gene Codes Corporation, Ann Arbor, MI, USA). Confirmed SNP sites in isotig sequences were used for CAPS marker development by SNP2CAPS [30]. For primer details and restriction enzymes see Additional file 4: Table S3.

The resistance gene candidate RGC151 had been identified earlier as co-segregating with the *Pl*_*ARG*_ locus [4]. One RGC151 corresponding isotig sequence (95% homology, E-value = 0.0), iso35499, was only identified in BR, thus it was not detected by the pipeline, since the sequence was not represented in BS. However, SNP detection in parental lines allowed for the development of a CAPS marker, and iso35499 was mapped as a control.

Finally, CAPS markers and SNP markers were integrated into the (cms)HA342 × ARG1575-2 genetic map [4] using JOINMAP 4.0 [31] with a LOD threshold > 3.0 and the Kosambi mapping function [32].

## Results

### Next generation sequencing and *de novo* transcriptome assembly

Normalized cDNA libraries of BR and BS were established for marker enrichment and identification of candidate genes for *Pl*_*ARG*_. The BRBS cDNA pool sequenced using the Roche 454 approach generated 2.53 million reads yielding a total of 857 Mb raw sequence data. After filtering low-quality reads and reads containing adapter sequences, 1,182,916 (426 Mb) and 1,177,524 reads (427 Mb) were obtained for BR and BS, respectively. Finally, 1,981,006 reads (83.9%) were assembled to a sunflower transcriptome reference (BRBS) of 54.9 Mb length (Table 1 and Additional file 5: Text S1). The assembly comprising 46,881 isotigs longer than 499 nt, resulted in 38,768 isogroups, 53,541 isotigs, 35,139 large contigs, and 133,795 singletons (Table 2). Singletons were excluded from further analysis. According to the description of the Newbler software, isogroups, isotigs and contigs approximately represent gene models, transcripts and exons (Additional file 3: Figure S1). On average, isogroups were built from 1.6 contigs and isotigs from 1.9 contigs. Since there are isogroups containing only one contig reflecting single exon genes, the average number of contigs in isogroups is lower than in isotigs. The largest isogroup and the largest isotig consisted of 50 and 15 contigs, respectively. The average size of isotigs was 1,077 nt, with a L50 length of 1, 296 nt and a maximum length of 14,431 nt. The average number of reads per isotig was 66.92, with 33.34 reads from BR and 33.58 reads from BS. Interestingly, 2,262 isotigs were assembled either only by reads from BR or BS, indicating that these isotigs represent most probably genes that were present only in BR or BS. Out of these 2,262 isotigs, 581 (25.7%) BS-only isotigs and 617 (27.3%) BR-only isotigs were assembled by more than five reads.

**Table 1:** Read statistics of 454 cDNA sequences after quality filtering

**Reads**	**Number of reads**	**Bases**
BR	1,182,916	425,618,400
BS	1,177,524	426,564,503
Aligned*	2,200,571	785,888,510
	(93.2%)	(92.2%)
Assembled*	1,981,006	54,877,004
Partially aligned*	219,291	-
Singletons*	133,795	-
Repeats*	471	-
Outliers*	24,280	-

*Definitions of these Newbler *de novo* assembly terms can be found in Additional file 5: Text S1.

Sequence libraries of one resistant bulk (BR) and one susceptible bulk (BS) each containing pooled cDNA of 16 individual F_2_ plants were generated to perform the 454 sequencing.

**Table 2:** Transcriptome assembly metrics

	**Isogroups**	**Category**	**Large contigs (>499 nt)**
		**Isotigs**	
Number	38,768	53,541	35,139
Average read number	N/A	66.92	N/A
Average contig number	1.6	1.9	N/A
Maximum contig number	50	15	N/A
Average size [nt]	N/A	1,077	997
L50 size [nt]	N/A	1,296	1,079
Largest size [nt]	N/A	14,431	9,147

A sunflower transcriptome reference assembly (BRBS) was developed by combining reads from the resistant bulk (BR) and the susceptible bulk (BS).

### SNP discovery

After independent alignment of BR and BS reads to the transcriptome reference sequence, SNP calling with SAM tools resulted in 2,085,664 SNPs. Isogroups carrying one or more SNPs were subsequently filtered for the read depth (≥ 5 reads at the SNP site each in BR and BS) and for distinctive SNP patterns as expected in BSA, meaning that SNPs should be homozygous for one allele in BR and homozygous for the alternate allele in BS. According to these criteria, SNPs were detected in 219 out of 38,768 isogroups and were functionally categorized using BLAST2GO (Additional file 6: Table S4). Besides 109 sequences that encoded proteins with unknown biological function or gave no hit, the sequences were assigned to the functional classes signal transduction (26), metabolism (22), transport (15), cell rescue, defense and ageing (15), protein processing (12), transcription (10), cellular biogenesis (4), energy (4), and protein synthesis (2) (Additional file 7: Figure S2).

The 219 isogroups were searched for similarity to a database containing 113 manually curated *R* genes. In total, 42 isogroup sequences were identified as putative resistance gene candidates. Almost all candidate genes showed sequence homology to the protein class of kinases. Twelve were similar to receptor-like kinases (RLK), and 11 sequences showed homology to the coiled-coil (CC)-NBS-LRR (CNL) subclass of R proteins. Five sequences were similar to R proteins that consist of a leucine-rich receptor-like repeat (RLP) and a short cytoplasmic region with no kinase domain. Out of each of the 42 isogroups that represent resistance gene candidates showing polymorphisms between BR and BS, one isotig was randomly selected for experimental SNP validation and subsequent integration of markers into the genetic map of (cms)HA342 x ARG1575-2 (Additional file 8: Table S5 and Additional file 9: Table S6).

### Genetic mapping of isotig sequences

We used KASPar assays and CAPS marker analysis for genotyping recombinant F_2_ individuals of the cross (cms)HA342xARG1575-2 with SNPs from the 42 isotigs. A summary of the SNP analysis is given in Table 3. Using flanking sequences of at least 50 bases on both sides of each SNP, an *in silico* KASPar assay was designed, which yielded eight SNPs for further processing. For CAPS marker analysis, PCR primers were designed for the remaining 34 isotigs from which 21 sequences were successfully amplified in the parental lines ARG1575-2 and HA342. Sanger re-sequencing of the 21 PCR amplicons confirmed the isotig sequences of the assembled 454 reads and allowed for SNP confirmation. When comparing sequences of ARG1575-2 to those of HA342 to confirm polymorphisms identified between the resistant and susceptible bulk based on NGS data, SNPs identified from 454 sequences were validated for six isotigs. Sanger re-sequencing in ARG1575-2 and HA342 did not confirm the *in silico* discovered SNPs of 15 isotigs. To map each of the six resistance gene candidates for which isotig polymorphisms were confirmed, one SNP per isotig was converted into a CAPS marker.

**Table 3:** Summary of SNP marker analysis

	**KASPar**	**CAPS**	**Total**
Primer design successful	8	21	29
SNPs confirmed	8	6	14
SNPs mapped	7	5	12

Proceeding from 42 isotigs, the outcome of SNP validation and map integration is shown for two genotyping platforms.

**Figure 2:**
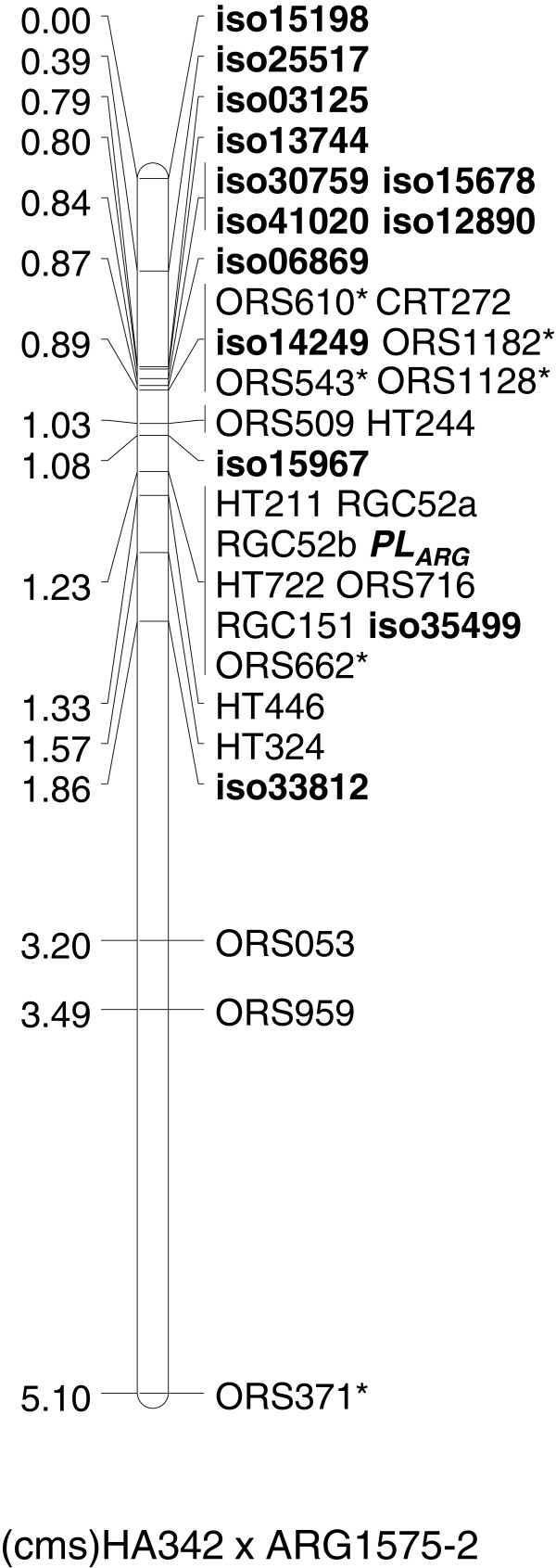
**Partial genetic map of (cms)HA342 × ARG1575-2 linkage group (LG) 1 around *Pl***_***ARG***_*.* Markers with an asterisk were screened in all individuals, while all other markers were screened in recombinant lines only. Resistance locus *Pl*_*ARG*_ and candidate sequences are shown in bold. Cumulative map distances in centiMorgan (left) are based on the analysis of 2,141 F_2_ plants.

For twelve of the fourteen (8 + 6) isotigs with confirmed SNP sites both BS and BR were homozygous for the corresponding KASPar and CAPS markers and they mapped on LG1 as expected. Since they were not linked to other markers in that region, two isotigs (iso15562 and iso34182) could not be validated as candidate sequences for the target region. Ten markers, 6 KASPar and 4 CAPS markers, mapped at the top of LG1 but outside of the target interval, CAPS marker iso33812 mapped below the target interval. However, CAPS marker iso15967 mapped in the target interval 0.15 cM above *Pl*_*ARG*_ (Figure 2). Additionally, CAPS marker iso35499 (RGC151) which was analysed as a control, mapped in the target interval on LG1 and co-segregated with *Pl*_*ARG*_, as expected (Figure 2).

## Discussion

Isolation of candidate genes underlying specific phenotypes by map based cloning requires the identification of markers closely flanking the respective locus [33]. BSA was developed for rapid identification of markers linked to any specific gene or genomic region [7,34]. The central idea of BSA is to form DNA pools of plants that differentiate with regard to phenotype. Any polymorphic marker with clear differentiation of the two bulks will be closely linked to the respective phenotype. A comprehensive marker resource as well as a high resolution mapping population are prerequisites to fine map the target interval for map based cloning in order to identify candidate genes [35]. In the present study, we extended the BSA approach to analyze cDNA sequences instead of DNA-based molecular markers such as microsatellites, which are available only in limited numbers for the target region. The introduction of NGS technologies was a critical development to allow for such a massively parallel approach in a time- and cost efficient way. Using cDNAs as sequencing templates reduced data complexity compared to whole-genome sequencing and enabled marker discovery in expressed sequences that directly delivered candidates for the trait of interest. Our assumption was that *Pl*_*ARG*_ is constitutively expressed in non-inoculated plant material, thus we did not include RNA from infected tissue, as this has no impact on the development of the pipeline in general.

*De novo* transcriptome assembly is a computational challenge especially in plant genomes where multiple rounds of genome duplication events during evolution created paralogous copies of ancestral genes. Here, Newbler GS De Novo Assembler 2.5.3 (Roche, Branford, CT, USA) was used to create a sunflower transcriptome assembly (see Figure 1, part 1 and 2). This assembler performed very well or even best on 454 transcriptome data in a simulation study [36] as well as in a systematic comparison [16] of different software for *de novo* assembly. We cannot completely rule out the possibility of creating apparently heterozygous isotigs which result from assembly of paralogous sequences. As there is currently no computational solution that overcomes this specific transcriptome assembly problem, we relied on the experimental SNP verification step for obtaining the correct candidate genes. Our results proved the feasibility of this strategy.

Generally, the sequence alignment can be performed by different NGS alignment programs such as for example Bowtie, SOAP (Short Oligonucleotide Alignment Program), and the frequently used MAQ (Mapping and Assembly with Qualities) software. The BWA package was used for read alignment (see Figure 1, part 3) since it has been shown to reach similar accuracy but is at least ten times faster compared to MAQ [20]. It further allows for the implementation of SAMtools to extract alignments in a region, merge and sort alignments and to complete SNP calls [20,21]. In our study, only SNPs were considered as polymorphisms but the pipeline can be easily extended to call insertion/deletion polymorphisms.

In 13 out of 42 cases, the SNP validation assays failed at the design stage, either due to a high number of neighboring SNPs in the respective sequences or due to the lack of sufficiently long suitable flanking sequences for assay design. During the validation procedure, 15 out of 21 SNP containing isotigs were identified as false positives, meaning they were in fact monomorphic between the parental lines. In rare cases, this might be the result of mapping paralogs to the reference sequence, if simultaneously homologous sequences are not present at all in the same bulk. *R* gene classes often consist of several highly similar family members and often the genes are clustered in the same genomic region [37-40]. Meyers *et al.*[41] analyzed a resistance gene cluster in lettuce spanning at least 3.5 Mb. They observed a nucleotide identity of 53–97% between members of the *RGC2* gene family. But the more likely explanation for the high number of false positives is an insufficient sequence coverage that results in undiscovered heterozygous states of the bulks.

Two of the fourteen isotigs (iso15562 and iso34182) could not be confirmed as candidate genes for the target region by genetic mapping. During SNP calling, SNPs were identified with at least 5-fold sequence coverage in both bulks. Coverage in the sequence assemblies of iso34182 with 5/5 (BR/BS) reads per SNP was at the lower limit and might have been too low to provide high-confidence SNPs. Nielsen *et al.* recommend a >20-fold coverage to achieve a sufficient reliability of SNP data [42]. However, applying the very stringent criterion of 10/10 (BR/BS) reads per SNP would have resulted in a loss of seven of the twelve new SNP markers in our case. For iso15562 and iso34182, the DNA pools of both bulks in the experimental validation were heterozygous for the corresponding SNP markers. Since all plants constituting the bulks were chosen to be homozygous in the target interval as determined by the molecular marker genotypes these two SNPs could not be assigned to the target interval on LG1.

As expected, the control iso35499 which corresponds to RGC151 co-segregated with *Pl*_*ARG*_. This isotig has been assembled only by reads from BR. Reads from BS covering that region were not present in our dataset, probably due to insufficient sequencing depth. This can be concluded from the fact that from genomic DNA of both parents the sequence can be amplified as well as from the observation that the sequence is expressed at low level also in the susceptible parent [15]. With decreasing costs for sequencing, it should be feasible in future studies to increase sequence coverage and set more stringent coverage thresholds during SNP calling to avoid false positives.

For our purpose, an ideal bulk would contain genotypes that are homozygous in the target interval defined by the markers flanking the *Pl*_*ARG*_ locus, with each plant carrying a recombination event on either side of that region for sharply delimiting the target interval. Out of the 14 isotigs for which the polymorphisms between the two bulks were confirmed, 12 candidate sequences were mapped to LG1. F_2_ plants assigned to the susceptible bulk BS did not show a recombination above the resistance locus (Additional file 2: Table S2) resulting in mapping of 10 isotigs outside the target interval. Iso15967 mapped between the markers delimiting the target interval as anticipated. The identification of iso15967 as a new closely linked flanking marker to *Pl*_*ARG*_ narrows the target interval from 0.30 cM [4] to 0.25 cM in the (cms)HA342 x ARG1575-2 population.

Non-coding DNA regions are less conserved than coding sequences. For that reason the expected SNP number is lower when using transcriptome data for SNP discovery instead of genomic sequences.

Our strategy yielded 12 polymorphic SNPs in *R* gene like sequences that could be used to fine map LG1. The SNP detection pipeline developed for this study had a conversion rate of 33% validated SNPs (14 out of 42) which was lower than in other reports [13,43,44]. The lower conversion rate was in part attributed to the high number of consecutive polymorphisms within SNP flanking sequences (Additional file 8: Table S5). Here, 2,085,664 SNPs were detected in total, regardless of sequence coverage and alignment quality. Based on this number, we calculated an upper limit of 1 SNP/26 bp as SNP frequency in the transcriptome reference sequence. For cultivated sunflower a SNP density of ~ 1 SNP/65 bp has been reported [45,46]. The high SNP density in our dataset may result from the interspecific sequence comparison between *H. annuus* and *H. argophyllus* (the donor of the *Pl*_*ARG*_ locus) in the target region. This extraordinary sequence polymorphism together with insufficient length of candidate sequences prohibited the successful assay design for 13 out of 42 SNPs (31%). Finally, out of 29 candidates 14 SNPs have been confirmed which corresponds to a SNP confirmation rate of 48%.

Sequencing with deeper coverage can improve our method by 1) increasing the probability of an *in silico* detected SNP being a truly polymorphic site, 2) providing sufficient flanking sequence information surrounding the SNP that is necessary for experimental SNP validation. The establishment of bulks with equally distributed recombination events on both sides of the target interval would further increase the number of true candidate sequences which map inside the target interval. Moreover, to enhance the chances for marker enrichment the procedure could be extended to the facultative transcriptome by using pathogen infected plant material as source for cDNA libraries.

We have shown that mining for desired gene classes is possible, but our approach is not limited to resistance genes. With respect to further marker enrichment, our future work will focus on the whole set of the 219 candidates which represent SNPs from expressed genes in the target region. Two other resources for region-specific markers will be exploited. First, the bioinformatics pipeline will be adjusted to enable Indel calling. Second, the 617 isotigs containing only BR sequences will be filtered for *R* gene-like sequences. As they could be potential candidate genes for *Pl*_*ARG*_ expressed only by lines carrying the corresponding gene from the resistant parent, the resulting candidate sequences will be analysed in detail.

In summary, BSTA allows for SNP marker enrichment in a specific genomic region by generating genome wide transcriptome sequence information. Depending on the RNA source, the transcriptome can be covered at adequate sequence coverage through deep sequencing. Thus, a large sequence resource is produced that can be valorized by mining SNPs in any expressed gene or for identifying candidate genes in any desired target region. The only prerequisite to identify candidate genes is that the genes of interest are expressed in the sample tissues. Resistance genes of the NBS-LRR type generally fulfill this requirement [14]. Regarding sequencing and *de novo* assembly, our approach is tailored to transcriptome analysis, but the work plan depicted in Figure 1 is applicable to bulked segregant whole genome analysis using appropriate sequencing technology and respective bioinformatics tools. As the additional Indel-calling, such an extension of SNP discovery to non-coding regions would be useful regarding functional analysis of genes such as transcription factor binding sites in promotor regions or loss-of-function due to insertion/deletion mutations.

## Conclusions

Our results show the potential of applying the BSTA approach for the identification of expressed genes in a target map interval. We focused here on sequences with homology to disease resistance genes, but the approach can be used for efficient enrichment of SNP markers in any target interval defined by appropriate phenotypic bulks. To maximize the accuracy and prevent false-positive SNP detection, sufficient sequence coverage is necessary and stringent criteria have to be applied to the pipeline. We demonstrated the feasibility of the BSTA approach for marker enrichment and fine-mapping of specific genomic regions in a non-model organism.

## Availability of supporting data

The data sets supporting the results of this article are available in the GABI primary database repository, http://www.gabipd.org/download/cgi-bin/Download.pl.cgi.

## Competing interests

The authors declare that they have no competing interests.

## Authors’ contributions

EB, SK, CCS and SW conceived and designed the experiments; VH developed the mapping population; SW and VH carried out resistance tests; ST conducted the 454 sequencing; YW designed the bioinformatics pipeline; YW and ML carried out sequence analysis; MS performed bioinformatics analyses in the initial stage of the project; ML and SW carried out the SNP validation and the genetic mapping; ML, YW, SW, GH, SK, CCS and EB, drafted and revised the manuscript. All authors read and approved the final version of the manuscript.

## Supplementary Material

Additional file 1: Table S1 Composition of the resistant bulk BR. Graphical genotypes of BR containing 16 progenies in (cms)HA342xARG1575-2 and their marker scores are shown. Abbreviations are explained at the end of the table. Click here for file

Additional file 2: Table S2 Composition of the susceptible bulk BS. Graphical genotypes of BS containing 16 progenies in (cms)HA342xARG1575-2 and their marker scores are shown. Abbreviations are explained at the end of the table. Click here for file

Additional file 3: Figure S1 Transcriptome assembly. Relationship between isogroups, isotigs and exons according to Nederbragt 2010 [17]. During transcriptome assembly, Newbler builds contig graphs. Reads coming from the transcript of a certain gene will result in a single contig graph. However, splice-variants will result in reads that have an insert representing an additional exon, which can cause a break the contig graph. Subsequently, there may be several contigs for each transcript, which themselves form a small contig graph. Thus, there will be numerous subgraphs, which are named isogroups by Newbler, each potentially representing one gene. To generate transcript variants, Newbler will traverse the contigs in the subgraphs of each isogroup, which are called isotigs. The isotigs represent alternative splice-variants, and the contigs represent the exons of a gene. Click here for file

Additional file 4: Table S3 List of PCR primers and restriction enzymes for CAPS marker analysis. Primers designed on *de novo* BRBS assembly of cDNA sequences derived from the susceptible (BS) and the resistant bulk (BR). Marker names, primer pair sequences, and expected fragment sizes are given. Additionally, restriction enzymes used for CAPS marker analysis are listed. Click here for file

Additional file 5: Text S1 Definitions of assembly terms. Definitions of Newbler *de novo* assembly terms for read statistics of 454 cDNA sequences after quality filtering. Click here for file

Additional file 6: Table S4 Annotation details. Functional annotation of 219 sequences with distinctive SNP patterns using the Blast2GO suite. Click here for file

Additional file 7: Figure S2 Schematic view on functional sequence annotation. Functional categorization of 219 candidates with distinctive SNP patterns into cellular processes derived from BLAST2GO sequence annotation. Sequences without annotation (no hit) or with unidentified biological function (unknown) are also included in this figure. Click here for file

Additional file 8: Table S5 Blast analysis. Summary of tblastx analysis for 219 candidate sequences against 113 manually curated *R* genes. Click here for file

Additional file 9: Table S6 SNP details. SNP positions and alleles of the 42 resistance gene-like candidate sequences. Click here for file
