# Supplementary material for: BSTA: a targeted approach combines bulked segregant analysis with next- generation sequencing and de novo transcriptome assembly for SNP discovery in sunflower
Source: BMC Genomics. 2013 Sep 17;14:628. doi: 10.1186/1471-2164-14-628 (PMC3848877; doi:10.1186/1471-2164-14-628)
Supplement: Additional file 1: Table S1 — Composition of the resistant bulk BR. Graphical genotypes of BR containing 16 progenies in (cms)HA342xARG1575-2 and their marker scores are shown. Abbreviations are explained at the end of the table. [file 1471-2164-14-628-S1.pdf]

Table S1 – Composition of the resistant bulk BR

Graphical genotypes of BR containing 16 progenies in (cms)HA342xARG1575-2 and their marker scores are shown. Abbreviations are explained at the end of the table.

								**Target region**									
	F2/F3 progeny	CRT 272	ORS 543	ORS 1128	ORS 1182	ORS 610	ORS 509	ORS 662	ORS 716	*Pl*$_{ARG}$	RGC 151	HT 722	HT 446	HT 324	ORS 053	ORS 959	ORS 371
**1**	4001-016	B	B	B	B	B	B	B	B	B	B	B	B	B	H	H	H
**2**	4004-033	B	B	B	B	B	B	B	B	B	B	B	B	B	H	H	H
**3**	4005-112	H	H	H	H	B	B	B	B	B	B	B	B	B	B	B	B
**4**	4005-155	B	B	B	B	B	B	B	B	B	B	B	B	H	H	H	H
**5**	4006-012	H	H	H	H	B	B	B	B	B	B	B	B	B	B	B	B
**6**	4006-072	B	B	B	B	B	B	B	B	B	B	B	B	B	H	H	H
**7**	4007-083	B	B	B	B	B	B	B	B	B	B	B	B	B	H	H	H
**8**	4007-096	B	B	B	B	B	B	B	B	n.d.	B	B	B	B	H	H	H
**9**	697	B	B	B	B	B	B	B	B	n.d.	B	B	B	B	H	H	H
**10**	781	B	B	B	B	B	B	B	B	B	B	B	B	B	H	H	H
**11**	1071	B	B	B	B	B	B	B	B	B	B	B	B	B	H	H	H
**12**	1092	B	B	B	B	B	B	B	B	n.d.	B	B	B	B	H	H	H
**13**	2473	B	B	B	B	B	B	B	B	B	B	B	B	B	H	H	H
**14**	2304	B	B	B	B	B	B	B	B	B	B	B	A	A	A	A	A
**15**	2105	A	A	A	A	A	A	B	B	B	B	B	B	n.d.	B	B	B
**16**	2313	A	A	A	A	A	A	B	B	B	B	B	B	B	B	B	B

yellow/ A: (cms)HA342 allele green / B: ARG1575-2 allele grey / H: heterozygous

n.d.: not determined
