# Supplementary material for: BSTA: a targeted approach combines bulked segregant analysis with next- generation sequencing and de novo transcriptome assembly for SNP discovery in sunflower
Source: BMC Genomics. 2013 Sep 17;14:628. doi: 10.1186/1471-2164-14-628 (PMC3848877; doi:10.1186/1471-2164-14-628)
Supplement: Additional file 2: Table S2 — Composition of the susceptible bulk BS. Graphical genotypes of BS containing 16 progenies in (cms)HA342xARG1575-2 and their marker scores are shown. Abbreviations are explained at the end of the table. [file 1471-2164-14-628-S2.pdf]

Table S2 – Composition of the susceptible bulk BS

Graphical genotypes of BS containing 16 progenies in (cms)HA342xARG1575-2 and their marker scores are shown. Abbreviations are explained at the end of the table.

								Target region									
	F2 progeny	CRT 272	ORS 543	ORS 1128	ORS 1182	ORS 610	ORS 509	ORS 662	ORS 716	PI_{ARG}	RGC 151	HT 722	HT 446	HT 324	ORS 053	ORS 959	ORS 371
**1**	4006-056	A	A	A	A	A	A	A	A	n.d.	A	A	A	H	H	H	H
**2**	4007-006	A	A	A	A	A	A	A	A	n.d.	A	A	A	H	H	H	H
**3**	4007-034	A	A	A	A	A	A	A	A	A	A	A	A	A	H	H	H
**4**	4007-126	A	A	A	A	A	A	A	A	A	A	A	A	H	H	H	H
**5**	4008-120	A	A	A	A	A	A	A	A	A	A	A	A	A	H	H	H
**6**	4008-195	A	A	A	A	A	A	A	A	n.d.	A	A	A	A	H	H	H
**7**	4010-007	A	A	A	A	A	A	A	A	A	A	A	A	A	H	H	H
**8**	4010-034	A	A	A	A	A	A	A	A	n.d.	A	A	A	A	H	H	H
**9**	4010-199	A	A	A	A	A	A	A	A	A	A	A	A	A	H	H	H
**10**	390	A	A	A	A	A	A	A	A	A	A	A	A	A	H	H	H
**11**	1010	A	A	A	A	A	A	A	A	A	A	A	A	A	H	H	H
**12**	1343	A	A	A	A	A	A	A	A	A	A	A	A	A	H	H	H
**13**	1731	A	A	A	A	A	A	A	A	A	A	A	A	A	H	H	H
**14**	2127	A	A	A	A	A	A	A	A	A	A	A	A	A	H	H	H
**15**	2245	A	A	A	A	A	A	A	A	A	A	A	A	H	H	H	H
**16**	2394	A	A	A	A	A	A	A	A	A	A	A	A	A	H	H	H

yellow / A: (cms)HA342 allele   grey / H: heterozygous   n.d.: not determined
