# Supplementary figures and images for: BSTA: a targeted approach combines bulked segregant analysis with next- generation sequencing and de novo transcriptome assembly for SNP discovery in sunflower
Source: BMC Genomics. 2013 Sep 17;14:628. doi: 10.1186/1471-2164-14-628 (PMC3848877; doi:10.1186/1471-2164-14-628)

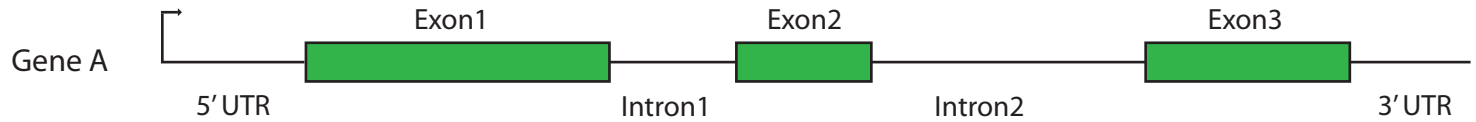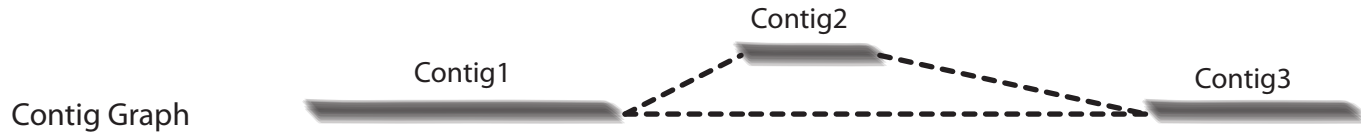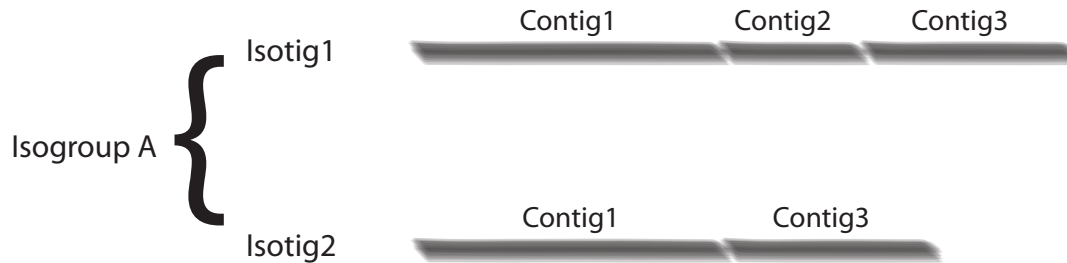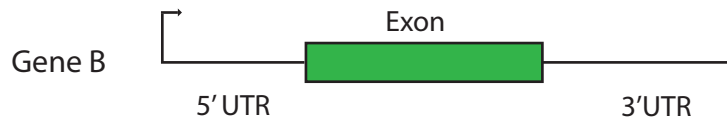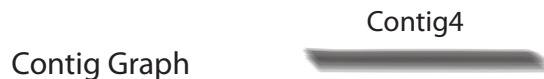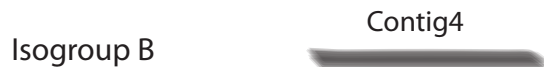

Supplement: Additional file 3: Figure S1 — Transcriptome assembly. Relationship between isogroups, isotigs and exons according to Nederbragt 2010 [17]. During transcriptome assembly, Newbler builds contig graphs. Reads coming from the transcript of a certain gene will result in a single contig graph. However, splice-variants will result in reads that have an insert representing an additional exon, which can cause a break the contig graph. Subsequently, there may be several contigs for each transcript, which themselves form a small contig graph. Thus, there will be numerous subgraphs, which are named isogroups by Newbler, each potentially representing one gene. To generate transcript variants, Newbler will traverse the contigs in the subgraphs of each isogroup, which are called isotigs. The isotigs represent alternative splice-variants, and the contigs represent the exons of a gene. [file 1471-2164-14-628-S3.pdf]
