# Supplementary material for: BSTA: a targeted approach combines bulked segregant analysis with next- generation sequencing and de novo transcriptome assembly for SNP discovery in sunflower
Source: BMC Genomics. 2013 Sep 17;14:628. doi: 10.1186/1471-2164-14-628 (PMC3848877; doi:10.1186/1471-2164-14-628)
Supplement: Additional file 4: Table S3 — List of PCR primers and restriction enzymes for CAPS marker analysis. Primers designed on de novo BRBS assembly of cDNA sequences derived from the susceptible (BS) and the resistant bulk (BR). Marker names, primer pair sequences, and expected fragment sizes are given. Additionally, restriction enzymes used for CAPS marker analysis are listed. [file 1471-2164-14-628-S4.pdf]

Table S3 – List of PCR primers and restriction enzymes for CAPS marker analysis

Primers designed on *de novo* BRBS assembly of cDNA sequences derived from the susceptible (BS) and the resistant bulk (BR). Marker names, primer pair sequences, and expected fragment sizes are given. Additionally, restriction enzymes used for CAPS marker analysis are listed.

Marker name	Forward primer (5'-3')	Reverse primer (5'-3')	Expected fragment size (bp)	Restriction enzyme
iso06869	TGGATCGTGTTCTTGCTCTCT	CGACACATTACTCGCCAAACT	707	*Nci*I
iso14249	CTGCAATTTTAGACCCGGTAGAT	GGTTCAAGCTCAGGTCAAGATAGT	684	*BstN*I
iso15198	GGAACCCGGTCTGCAATAC	CCGAGAAATCATACGGCATT	465	*Mbo*II
iso15967	CACAAACCGTGTCATAGACTTTTC	AGCCTTCTGATCGATACTTGAGTT	746	*BstU*I
iso33812	TACCCGAGGGTATGTTTGAGC	GCTTGGAAATATGCCTCTATCG	1300	*Hpa*II
iso35499	AGCGTTGTTGATGCACTGTT	ATGCCCGATTGTGTGAAAAG	674	*Aci*I
