# Supplementary material for: BSTA: a targeted approach combines bulked segregant analysis with next- generation sequencing and de novo transcriptome assembly for SNP discovery in sunflower
Source: BMC Genomics. 2013 Sep 17;14:628. doi: 10.1186/1471-2164-14-628 (PMC3848877; doi:10.1186/1471-2164-14-628)
Supplement: Additional file 5: Text S1 — Definitions of assembly terms. Definitions of Newbler de novo assembly terms for read statistics of 454 cDNA sequences after quality filtering. [file 1471-2164-14-628-S5.pdf]

Text S1 ー Definitions of Newbler *de novo* assembly terms for read statistics of 454 cDNA sequences after quality filtering (see Table 1).

Aligned - the number of reads and bases that were aligned to other reads

Assembled - the read is fully incorporated into the assembly

Partially Aligned - only part of the read was included in the assembly, the rest was deemed to have diverged sufficiently to not be included

Singleton - the read did not overlap with any other reads in the input

Repeat - the read was either:

> i. Inferred to be repetitive early in the assembly process. A read can be inferred to be repetitive if >70 % of the read's seeds hit to at least 70 other reads. Such reads are excluded from the assembly.
>
> ii. Determined to partially overlap a contig. The portions of such reads that overlap unique contigs are still included in the assembly results.

Outlier – the read was identified by the GS De Novo Assembler as problematic, and was excluded from the final contigs (one explanation of these outliers are chimeric sequences, but sequences may be identified as outliers simply as an assembler artefact).
