# Supplementary material for: BSTA: a targeted approach combines bulked segregant analysis with next- generation sequencing and de novo transcriptome assembly for SNP discovery in sunflower
Source: BMC Genomics. 2013 Sep 17;14:628. doi: 10.1186/1471-2164-14-628 (PMC3848877; doi:10.1186/1471-2164-14-628)
Supplement: Additional file 7: Figure S2 — Schematic view on functional sequence annotation. Functional categorization of 219 candidates with distinctive SNP patterns into cellular processes derived from BLAST2GO sequence annotation. Sequences without annotation (no hit) or with unidentified biological function (unknown) are also included in this figure. [file 1471-2164-14-628-S7.pptx]

## Slide 1
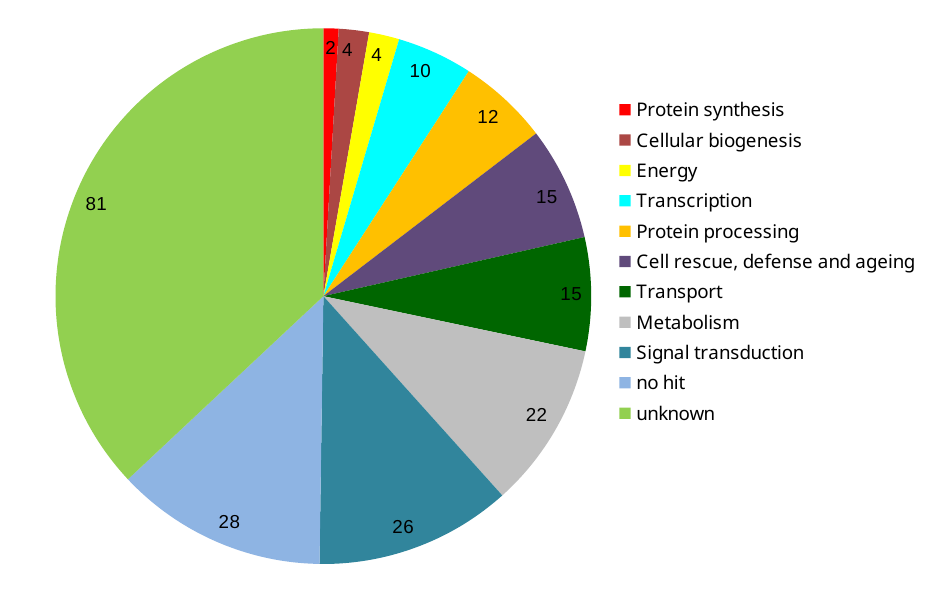

### Chart
| Category | |
|---|---|
| Protein synthesis | 2.0 |
| Cellular biogenesis | 4.0 |
| Energy | 4.0 |
| Transcription | 10.0 |
| Protein processing | 12.0 |
| Cell rescue, defense and ageing | 15.0 |
| Transport | 15.0 |
| Metabolism | 22.0 |
| Signal transduction | 26.0 |
| no hit | 28.0 |
| unknown | 81.0 |
